# Trends in gynaecologic cancer mortality and the impact of the COVID-19 pandemic in the United States

**DOI:** 10.1186/s13027-024-00567-6

**Published:** 2024-02-20

**Authors:** Yuyan Xi, Yuxin Guo, Sikai Qiu, Fan Lv, Yujiao Deng, Jingyi Xie, Zixuan Xing, Yajing Bo, Chenyu Chang, Fan Zhang, Fanpu Ji, Mu Li

**Affiliations:** 1 Department of Gynecology and Obstetrics, Second Affiliated Hospital of Xi’an Jiaotong University, No.157 Xi Wu Road, Xi’an, Shaanxi 710004 China; 2 Department of Infectious Diseases, Second Affiliated Hospital of Xi’an Jiaotong University, No.157 Xi Wu Road, Xi’an, Shaanxi 710004 China; 3 Second Affiliated Hospital of Xi’an Jiaotong University, Xi’an, China; 4 School of Mathematics and Statistics, Xi’an Jiaotong University, Xi’an, China; 5 Department of Gastroenterology, Second Affiliated Hospital of Xi’an Jiaotong University, Xi’an, China; 6 Northwest Women’s and Children’s Hospital, Xi’an, China; 7 National and Local Joint Engineering Research Center of Biodiagnosis and Biotherapy, Second Affiliated Hospital of Xi’an Jiaotong University, Xi’an, Shaanxi China; 8 Shaanxi Provincial Clinical Medical Research Center of Infectious Diseases, Xi’an, China; 9 Key Laboratory of Surgical Critical Care and Life Support (Xi’an Jiaotong University), Ministry of Education, Xi’an, China; 10 Key Laboratory of Environment and Genes Related to Diseases, Xi’an Jiaotong University, Ministry of Education of China, Xi’an, China

**Keywords:** Mortality, Ovarian cancer, Cervical cancer, Uterine corpus cancer, COVID-19

## Abstract

**Objectives:**

Our aim was to assess the trend in gynaecologic cancer (GC) mortality in the period from 2010 to 2022 in the United States, with focus on the impact of the pandemic on increased deaths.

**Methods:**

GC mortality data were extracted from the Center for Disease Control and Prevention Wide-Ranging Online Data for Epidemiologic Research (CDC WONDER) platform. We analysed mortality trends and evaluated observed vs. predicted mortality for the period from 2020 to 2022 with joinpoint regression and prediction modelling analyses.

**Results:**

A total of 334,382 deaths among adults aged 25 years and older with gynaecologic cancer were documented from 2010 to 2022. The overall age-standardised mortality rate (ASMR, per 100,000 persons) for ovarian cancer-related death decreased gradually from 7.189 in 2010 to 5.517 in 2019, yielding an APC (annual percentage change) of -2.8%. However, the decrease in ovarian cancer-related mortality slowed down by more than 4-fold during the pandemic. Cervical cancer -related mortality decreased slightly prior to the pandemic and increased during the pandemic with an APC of 0.6%, resulting in excess mortality of 4.92%, 9.73% and 2.03% in 2020, 2021 and 2022, respectively. For uterine corpus cancer, the ASMR increased from 1.905 in 2010 to 2.787 in 2019, and increased sharply to 3.079 in 2021 and 3.211 in 2022. The ASMR rose steadily between 2013 and 2022, yielding an APC of 6.9%.

**Conclusions:**

Overall, we found that GC-related mortality increased during the COVID-19 pandemic, and this increase was not specific to age, race, or ethnicity.

**Supplementary Information:**

The online version contains supplementary material available at 10.1186/s13027-024-00567-6.

## Introduction

Gynaecologic cancer (GC) continues to be a significant disease burden globally and in the United States. Ovarian cancer (OC) is the most lethal of all malignancies found in women. Even though the mortality rate for OC is decreasing remarkably [1], the prognosis is often poor due to asymptomatic or surreptitious growth of the tumour, with a 5-year relative survival rate of 49% [2]. As the second most common cancer in less developed regions, cervical cancer (CC) ranks eleventh in more developed regions [3]. Since 2006, human papilloma virus (HPV) vaccination has been recommended for young females aged 11–26 years in the U.S.; this led to a 43.35% decrease in CC mortality among young females, despite a less substantial decrease in older women [4]. Furthermore, a recent study [5] noted rapid changes in GC landscape, with the risk of death from uterine corpus cancer (UCC) being similar to that for OC among women overall. As UCC is associated with the metabolic syndrome and fertility patterns, changes related to these factors brought about by the pandemic must be considered.

Since 2020, the global population has experienced several waves of the COVID-19 pandemic and remarkable associated excess mortality [6]. Several studies have analysed the excess of cancer-related mortality during the pandemic [6–8]. However, there is no study reporting the impact of the COVID-19 pandemic on GC-related mortality. In this study, we provide an updated summary of the mortality rates of ovarian, cervical, and uterine cancers in the United States, with special attention paid to disparities observed across age and race groups.

## Methods

### Study design and population

This study performs time series and predictive analyses of GC-related mortality rates in the U.S. population from 01/01/2010-12/31/2022. Data were obtained through the National Vital Statistics System (NVSS) dataset on the Center for Disease Control and Prevention Wide-Ranging Online Data for Epidemiologic Research (CDC WONDER) website. The database contains annual mortality data with respect to 99% of deaths across all states and the District of Columbia. Also, we collected demographic data including age, race, and cause of death for those records with death related to GC. Institutional Review Board (IRB) approval was not sought for this study, as data from the NVSS website were publicly available and fully deidentified. The study complies with the Strengthening the Reporting of Observational Studies in Epidemiology (STROBE) guidelines.

### Definitions

In NVSS dataset, Data on GC-related deaths were collected from 01/01/2010-12/31/2022 for people aged ≥ 25 years. Causes of death were recorded using the tenth edition of the International Classification of Diseases (ICD-10). Based on the scope of our study, the ICD-10 codes of GC-related death causes were C56 for OC, C53 for CC, C54 for UCC, and U07.1 for COVID-19. Tumour were not usually the direct cause of death in patients; therefore, to avoid bias, we defined GC-related deaths as those with gynaecologic tumours listed as the primary cause of death or the underlying cause of death. Age stratification was performed and three categories were established: 25–44 years; 45–64 years; and ≥ 65 years. Race and ethnicity groups were defined as Hispanic, non-Hispanic Alaska Indians/American Natives (AI/AN), non-Hispanic Asian, non-Hispanic black, and non-Hispanic white.

### Statistical analysis

Demographic characteristics of decedents with GC are presented as frequencies with percentages. The crude death rate (per 100,000 people) was calculated by taking the number of deaths for each type of GC and dividing it by the total U.S. population in the corresponding year. The age-standardised mortality rate (ASMR, per 100,000 people) was determined by multiplying the age-specific mortality rate by the number of people in each age group in the standard population. According to the 2000 U.S. Census Standard Population, the age structure is divided into groups of 10 years, beginning at 25 years and ending with the oldest age group of 85 years and older. To quantify GC-related mortality during the pandemic, we performed a predictive analysis based on the ASMR during 2010–2019 to determine expected mortality in 2020, 2021 and 2022, and then compared the observed and expected mortalities. The predictive analysis was performed by a polynomial model, in each subgroup, we experimented with first-degree, second-degree, and third-degree polynomials and selected the model that best fit based on the RMSE values. COVID-19-related excess death was calculated by the percentage of COVID-19 related ASMR in totally excess deaths.

Subgroup analysis was conducted by age, sex, and race/ethnicity. Sex-specific mortality rates were calculated within each age group. All analyses were carried out using the Joinpoint Trend Analysis software (version 4.9.1.0; National Cancer Institute, Bethesda, MD), PyCharm3.9.0 (prediction analysis), and R 4.0.2 software (all other analyses and data cleaning). A two-tailed *p*-value of 0.05 was used to determine significance.

## Results

### Decedent population and characteristics for GC

**Fig. 1:**
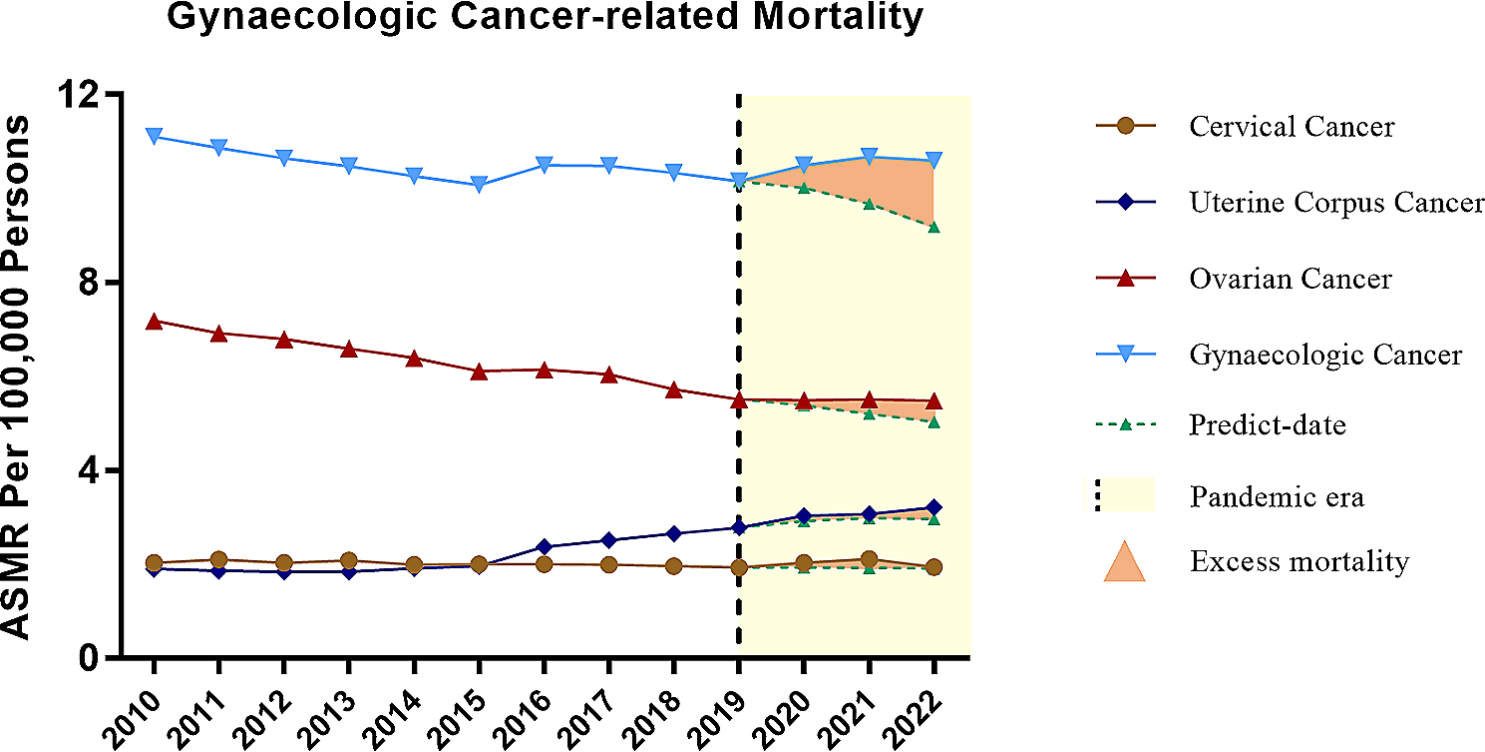
Age-standardised mortality rates for gynaecologic cancer in the U.S., 2010–2022

**Fig. 2:**
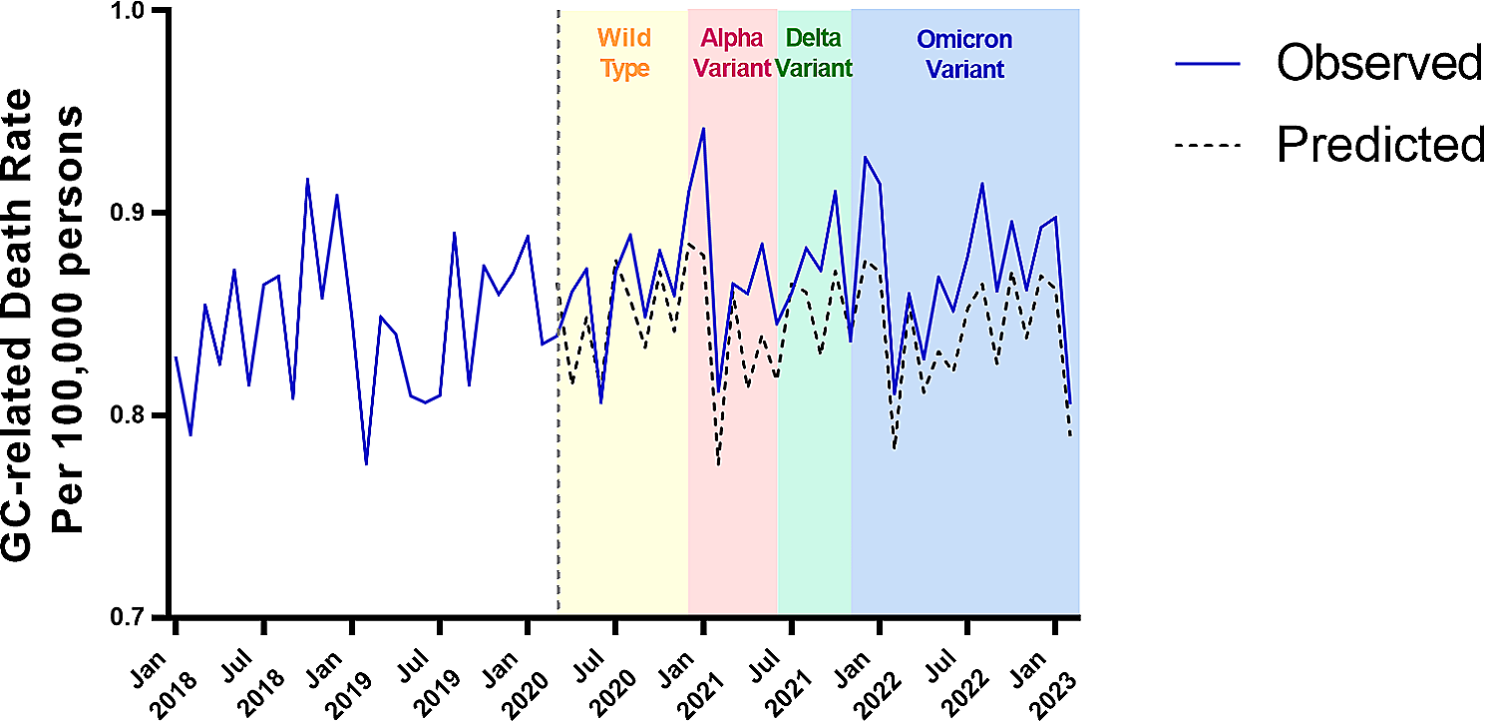
Age-standardised mortality rates for gynaecologic cancer in the U.S. during different phases of the COVID-19 pandemic, 2018–2022

A total of 334,382 deaths among adults aged 25 years and older with gynaecologic cancer were documented from 2010 to 2022. Ovarian cancer was the most common cause of death from gynaecological malignancy, accounting for 59.37% (195,885) of deaths, followed by UCC (79,143, 23.58%), and CC (60,540, 18.04%) (Table 1). The GC-related ASMR (per 100,000 persons) decreased gradually from 11.11 in 2010 to 10.16 in 2019, with only a mild increase in 2016. There was a rise in mortality in 2020 (10.49, 4.80% excess mortality) and a sustained increase in 2021 (10.68, 10.33% excess mortality) followed by a slight decline in 2022 (10.60, 15.37% excess mortality) (Fig. 1 and Table 2). Monthly ASMR mirrored patterns for different phases of the COVID-19 pandemic among women, particularly in the surge in deaths at the beginning of 2021 and 2022 (Fig. 2). Most decedents were ≥ 65 years old (63.04%), and were overwhelmingly non-Hispanic whites (75.29%) followed by non-Hispanic blacks (12.78%), and Hispanics (8.05%). However, non-Hispanic American Indians/Alaska Natives had the highest excess mortality of 34.33% (9.23 vs. 6.87 per 100,000 persons) (Table S1 and S2).

**Table 1:** Age characteristics of gynaecologic cancer deaths in the U.S., 2010–2022

		2010–2022	2010	2019	2020	2021	2022
Gynecologic Cancer†	**Overall**	334,382 [100.00]	23,937 [100.00]	26,649 [100.00]	27,969 [100.00]	28,154 [100.00]	28,017 [100.00]
	**Age**						
	25–44	17,367 [5.19]	1279 [5.34]	1315 [4.93]	1397 [4.99]	1540 [5.47]	1361 [4.86]
	45–64	106,225 [31.77]	8042 [33.60]	8093 [30.37]	8331 [29.79]	8220 [29.20]	8121 [28.99]
	≥ 65	210,790 [63.04]	14,616 [61.06]	17,241 [64.70]	18,241 [65.22]	18,394 [65.33]	18,535 [66.16]
Ovarian Cancer	**Overall**	195,885 [100.00]	15,548 [100.00]	14,570 [100.00]	14,822 [100.00]	14,807 [100.00]	14,597 [100.00]
	**Age**						
	25–44	5547 [2.83]	464 [2.98]	407 [2.79]	416 [2.81]	424 [2.86]	433 [2.97]
	45–64	58,481 [29.85]	4829 [31.06]	4193 [28.78]	4240 [28.61]	4119 [27.82]	4110 [28.16]
	≥ 65	131,857 [67.31]	10,255 [65.96]	9970 [68.43]	10,166 [68.59]	10,264 [69.32]	10,054 [68.88]
Cervical Cancer	**Overall**	60,540 [100.00]	4346 [100.00]	4673 [100.00]	4905 [100.00]	5075 [100.00]	4772 [100.00]
	**Age**						
	25–44	10,376 [17.14]	744 [17.12]	768 [16.43]	845 [17.23]	944 [18.60]	775 [16.24]
	45–64	26,722 [44.14]	2033 [46.78]	2038 [43.61]	2067 [42.14]	2095 [41.28]	1987 [41.64]
	≥ 65	23,442 [38.72]	1569 [36.10]	1867 [39.95]	1993 [40.63]	2036 [40.12]	2010 [42.12]
Uterine Corpus Cancer	**Overall**	79,143 [100.00]	4119 [100.00]	7506 [100.00]	8349 [100.00]	8393 [100.00]	8787 [100.00]
	**Age**						
	25–44	1507 [1.90]	74 [1.80]	141 [1.88]	139 [1.66]	187 [2.23]	158 [1.80]
	45–64	21,488 [27.15]	1207 [29.30]	1894 [25.23]	2067 [24.76]	2047 [24.39]	2076 [23.63]
	≥ 65	56,148 [70.94]	2838 [68.90]	5471 [72.89]	6143 [73.58]	6159 [73.38]	6553 [74.58]

Data are presented as *n* [%]; age is presented in years. †Gynaecologic cancer includes cervix uteri (International Statistical Classification of Disease and Related Health Problems, 10th revision [ICD-10] code C53), corpus uteri (ICD-10 code C54), and ovary (ICD-10 code C56)

**Table 2:** Age-standardised mortality rate among U.S. women with gynaecologic cancer by age group, 2010–2022

Age-standardized Mortality Rate (Per 100,000 Persons)														
	Age Years	Pre-Pandemic Referent Epoch 2010	Pre-Pandemic Referent Epoch 2019	Pandemic Epoch 1 2020				Pandemic Epoch 2 2021				Pandemic Epoch 3 2022		
				Observed	Predicted [95% CI]	%Increase*		Observed	Predicted [95% CI]	%Increase*		Observed	Predicted [95% CI]	%Increase*
Gynecologic Cancer†	Overall	11.109	10.160	10.491	10.011 [9.275–10.746]	+ 4.796		10.677	9.677 [8.300-11.054]	+ 10.333		10.596	9.185 [6.891–11.478]	+ 15.366
	**Age**													
	25–44 years	1.673	1.627	1.682	1.613 [1.549–1.676]	+ 4.254		1.882	1.605 [1.532–1.678]	+ 17.234		1.63	1.598 [1.515–1.681]	+ 1.819
	45–64 years	9.425	8.807	9.125	8.769 [7.775–9.763]	+ 4.060		8.968	8.476 [6.613–10.338]	+ 5.804		8.889	8.050 [4.949–11.151]	+ 10.427
	≥ 65 years	36.331	32.668	33.677	32.102 [30.010-34.195]	+ 4.905		34.432	30.998 [27.077–34.919]	+ 11.077		34.758	29.338 [22.809–35.867]	+ 18.474
Ovarian Cancer	Overall	7.189	5.517	5.497	5.381 [5.257–5.505]	+ 2.157		5.514	5.206 [5.064–5.348]	+ 5.924		5.483	5.031 [4.871–5.191]	+ 8.990
	**Age**													
	25–44 years	0.582	0.527	0.473	0.493 [0.410–0.576]	-4.124		0.473	0.481 [0.363–0.598]	-1.732		0.518	0.468 [0.308–0.627]	+ 10.713
	45–64 years	5.621	4.529	4.629	4.443 [3.889–4.997]	+ 4.178		4.411	4.233 [3.194–5.271]	+ 4.199		4.411	3.973 [2.243–5.702]	+ 11.018
	≥ 65 years	25.533	19.028	18.878	17.926 [16.801–19.052]	+ 5.309		19.350	16.632 [14.524–18.741]	+ 16.336		19.083	15.071 [11.560-18.582]	+ 26.618
Cervical Cancer	Overall	2.038	1.937	2.034	1.939 [1.892–1.985]	+ 4.921		2.112	1.925 [1.871–1.978]	+ 9.730		1.950	1.911 [1.851–1.971]	+ 2.025
	**Age**													
	25–44 years	0.936	0.945	1.045	0.906 [0.865–0.948]	+ 15.38		1.145	0.872 [0.813–0.931]	+ 31.346		0.900	0.833 [0.753–0.912]	+ 8.028
	45–64 years	2.457	2.396	2.457	2.385 [2.284–2.486]	+ 3.034		2.457	2.376 [2.260–2.491]	+ 3.428		2.357	2.367 [2.236–2.497]	-0.416
	≥ 65 years	3.898	3.471	3.624	3.452 [3.242–3.662]	+ 4.996		3.786	3.400 [3.160–3.640]	+ 11.356		3.711	3.348 [3.077–3.620]	+ 10.832
Uterine Corpus Cancer	Overall	1.905	2.787	3.033	2.925 [2.548–3.303]	+ 3.678		3.079	2.981 [2.274-3. 689]	+ 3.302		3.211	2.962 [1.784–4.139]	+ 8.421
	**Age**													
	25–44 years	0.109	0.209	0.209	0.169 [0.109–0.229]	+ 23.640		0.264	0.176 [0.108–0.245]	+ 49.519		0.209	0.184 [0.106–0.261]	+ 13.624
	45–64 years	1.386	1.982	2.139	2.037 [1.619–2.454]	+ 5.048		2.100	1.973 [1.190–2.756]	+ 6.449		2.161	1.824 [0.520–3.128]	+ 18.461
	≥ 65 years	7.052	10.282	11.264	10.888 [9.704–12.072]	+ 3.453		11.444	11.190 [8.972–13.409]	+ 2.271		12.141	11.230 [7.534–14.922]	+ 8.116

Age data is presented in years. NA, not applicable. †Gynaecologic cancer includes cervix uteri (International Statistical Classification of Disease and Related Health Problems, 10th revision [ICD-10] code C53), corpus uteri (ICD-10 code C54), and ovary (ICD-10 code C56). *Denotes the % increase from predicted to observed value

### OC-related mortality

The overall ASMR for OC-related death decreased gradually from 2010 (7.189) to 2019 (5.517), yielding an annual percent change (APC) of -2.8%, *p* < 0.001; 95% CI = [-3.1, -2.4]. However, this decrease slowed down by more than 4-fold, with an APC of -0.6% during the pandemic; 95%CI = [-2.5 to 1.3] (Fig. 1 and Table S3). The observed ASMRs for 2020–2022 were higher than predicted (5.50 vs. 5.38 for 2020, 5.51 vs. 5.21 for 2021, 5.48 vs. 5.03 for 2022) (Table 2). The ASMR was highest in older adults throughout the study period, while the elderly were also the subgroup with the most significant downward trend in mortality with an APC of -2.3% (95%CI = [-2.8 to -1.9]). This is compared to the APCs of -1.6% and − 2.0% in younger and middle-aged women, respectively (Table S4).

OC-related mortality decreased across all races/ethnicities, with the most dramatic APC decrease in non-Hispanic American Indians/Alaska Natives (-3.6%) followed by non-Hispanic whites (-2.7%), non-Hispanic blacks (-2.2%), Hispanics (-2.0%) and non-Hispanic Asians (-0.8%) (Table S2). Although mortality rates continue to be higher in white versus black women, this disparity is narrowing (Fig. S1B). In 2020, all racial/ethnic groups saw a difference between observed and expected mortality, with the largest excess mortality in Non-Hispanic AI/ANs (88.13%), followed by Non-Hispanic Asians (19.38%) (Table S1).

### CC-related mortality

**Fig. 3:**
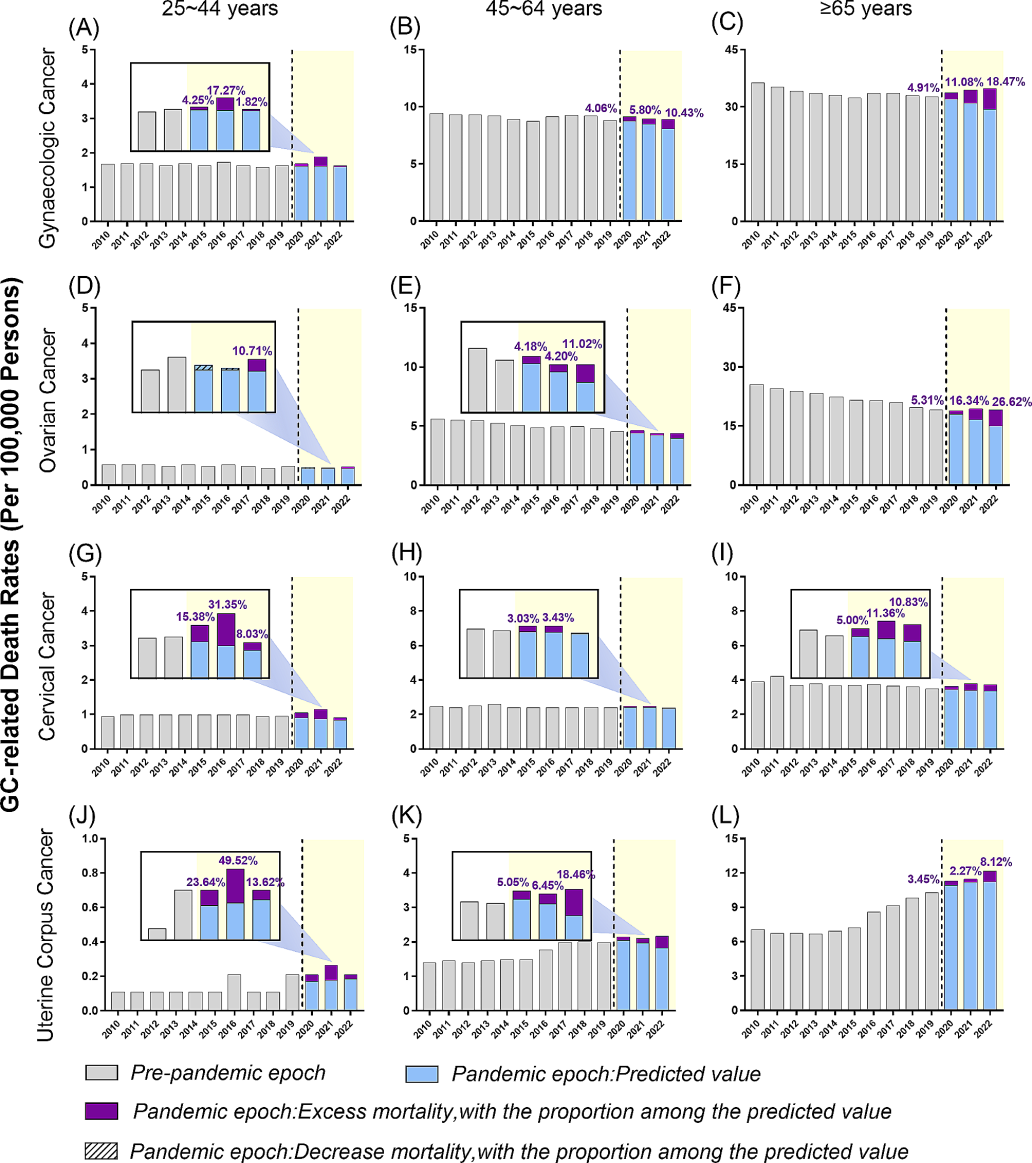
Age-standardized mortality rates for gynaecologic cancers in the U.S. in 2010–2022 by age group. (**A-C**): Gynaecologic cancer; (**D-F**): Ovarian cancer; (**G-I**): Cervical cancer; (**J-L**): Uterine corpus cancer. Gynaecologic cancer includes cervix uteri (International Statistical Classification of Disease and Related Health Problems, 10th revision [ICD-10] code C53), corpus uteri (ICD-10 code C54), and ovary (ICD-10 code C56)

Most decedents with cervical cancer were 45–64 years of age (44.14%) and greater than 65-years old (38.72%) at the time of death, while the proportion of deaths in younger people was also close to 20%. CC-related mortality slightly declined prior to the pandemic (APC − 0.6%), increased after 2018 (APC 0.6%) and peaked in 2021 (Table S3). Mortality trends stratified by age are shown in table S4. Notably, during the pandemic, the steepest rise in mortality was seen in older adults (APC 2.4%). Younger adults experienced a significant decrease in mortality ratio during the pandemic. However, this group exhibited the highest percentage of excess deaths (15.38% in 2020 and 26.13% in 2021) and showed a noticeable increase in 2021 (Fig. 3 and Table S4). The middle-aged group had a flat trend throughout the study period (APC − 0.2%) and relatively low excess mortality. In the older subgroup, about 30% of deaths were associated with SARS-CoV-2 infection (Table 3).

**Table 3:** The percentage of COVID-19 related in excess deaths in women with gynaecologic cancer in the U.S. by age group, 2020–2022

	Age Years	Pandemic Epoch 1 2020	Pandemic Epoch 2 2021	Pandemic Epoch 3 2022
		COVID%*	COVID%*	COVID%*
Gynecologic Cancer†	Overall	30.186	19.665	16.224
	**Age**			
	25–44 years	NA	0.000	187.562
	45–64 years	39.119	36.301	140.416
	≥ 65 years	31.551	20.164	13.453
Ovarian Cancer	Overall	57.465	32.196	18.404
	**Age**			
	25–44 years	NA	NA	NA
	45–64 years	21.166	56.260	8.975
	≥ 65 years	28.612	12.231	8.900
Cervical Cancer	Overall	13.217	17.640	85.373
	**Age**			
	25–44 years	NA	4.717	0.000
	45–64 years	0.000	48.236	--
	≥ 65 years	37.414	25.899	27.575
Uterine Corpus Cancer	Overall	43.844	62.892	26.744
	**Age**			
	25–44 years	NA	NA	NA
	45–64 years	38.213	30.880	11.667
	≥ 65 years	45.830	97.497	29.875

Abbreviation: NA: not applicable

†Gynecologic cancer includes cervix uteri (International Statistical Classification of Disease and Related Health Problems, 10th revision [ICD-10] code C53); corpus uteri (ICD-10 code C54); and ovary (ICD-10 code C56)

*Denotes the percentage of COVID-19 related ASMR in excess deaths

Values higher than 100 mean that the ASMR associated with COVID-19 is greater than the ASMR of excess deaths

The ASMR of cervical cancer decreased across all races and ethnicities during the study period, with the most dramatic APC decrease in non-Hispanic blacks (-2.4%, *p* < 0.001; 95%CI = [-3.1, -1.6]), followed by non-Hispanic AI/ANs (-2.2%), non-Hispanic Asians (-1.8%), and Hispanics (-0.9%) (Table S2). The high excess mortality due to CC during the pandemic was seen in Hispanics (18.88%) and non-Hispanic Asians (12.06%) (Table S1).

### UCC-related mortality

For uterine corpus cancer, the ASMR was stable between 2010 and 2013, then increased from 2013 onwards, yielding an APC of 6.9%, *p* < 0.001; 95%CI = [5.6, 8.3]. This continued in 2015 (Fig. 1), with increases in the mortality rate in the middle-aged subgroup (APC 10.7%, *p* < 0.05; 95%CI = [1.1 to 21.3]) and in the older subgroup (APC 11.5%, *p* < 0.05; 95%CI = [4.8 to 18.6]) (Table S4) during 2014–2017. As a result, the ASMR for uterine corpus cancer increased from 1.905 in 2010 to 2.787 in 2019, and further increased to 2.925 in 2020 and 3.074 in 2021 during the pandemic (Table 2). The excess mortality rates in uterine corpus cancer were 3.68% in 2020, 3.30% in 2021 and 8.42% in 2022. Of note, COVID-19-related deaths accounted for 43.84%, 62.89% and 26.74% of the excess deaths in 2020, 2021 and 2022, respectively (Table 2 and Table 3). The ASMRs stratified by age are shown in Fig. 3; Table 2. Importantly, the excess mortality in the younger subgroup was significantly higher than that in both the middle-aged and older subgroups during the COVID-19 pandemic (Table 2).

UCC-related mortality increased across all races/ethnicities through the study period, with an average APC of 7.0%, 6.1%, 4.8% and 4.6% in Hispanics, non-Hispanic blacks, non-Hispanic Asians, and non-Hispanic whites, respectively. The ASMR was roughly twice as high in non-Hispanic black women than that in women of other races/ethnicities (Fig. S1 and Table S1).

## Discussion

In this study, we assessed the temporal trends of mortality for three GCs (OC, CC, and UCC) from 2010 to 2022 to analyse the effect of the COVID-19 pandemic on GC-related mortality. We found an increase in GC-related mortality during each month between 2019 and 2022 that was significantly higher than predicted mortality based on pre-pandemic trends. There is already evidence that patients with gynaecological cancers may be particularly affected by the COVID-19 pandemic [9, 10]. A spike in growth was seen at the beginning of the prevalence of the Alpha and Omicron variant followed by a sharp decline. Considering the hysteresis of malignant tumor mortality, the relationship between this large fluctuation and different stages of COVID-19 requires longer studies. The overall curve for 2022 is downward, with the impact of COVID-19 likely to fade. Both OC- and CC-related mortality rates stably decreased prior to the pandemic, but this decrease slowed down in the former and reversed in the latter. For uterine corpus cancer, the ASMR rose steadily beginning in 2013, with a moderate rise after 2019, overall yielding an APC of 6.9% during 2013–2022. Importantly, we also found that effects of the pandemic on GC-related mortality are seen in all age groups, races, and ethnicities. However, there were marked disparities between subgroups.

COVID-19-related deaths account for 30% or lower of the excess deaths during the timeframe of the pandemic, emphasizing the notion that a non-COVID-19-related indirect increase in mortality deserves attention. Henley et al. [11] also found that an excess number of persons with cancer died from COVID-19 and other diseases than from underlying malignacy. Health care systems have experienced an unprecedented backlog of oncologic procedures, owing to a reduction in medical resources and screening programs as a result of shifting focus to care for an overwhelming number of COVID-19 patients. Patients were more likely to experience delays in the receipt of radiation therapy and less likely to undergo radiation therapy if adverse pathology was detected at the time of surgery [12]. Most affected was the young population, possibly owing to more barriers to treatment such as lack of transportation, financial strain, and scarcity of medical resources as a result of local epidemic prevention and control policies [13]. A mortality gap exists between non-Hispanic white and non-Hispanic black women for cervical and uterine cancers (and to a lesser extent for ovarian cancer). In 2010–2021, overall gyneacological cancer mortality is highest among the white population, largely due to the mortality rate of ovarian cancer and ageing of the population. Mortality rates of cervical and uterine cancers were higher in black women than in those of other races during the study period. This goes in line with known findings which showed that age-adjusted mortality rates in patients with cancer and COVID‐19 were found to be significantly higher in NH Black or African American, Hispanic or Latino, and NH American Indian or Alaskan Native groups in 2020 [14].

Ovarian cancer is the leading cause of death in middle-aged and older women. Young women are more likely to have low stage and low grade epithelial cancers [15], and they are able to tolerate more aggressive surgery and chemotherapy. In contrast, older patients, often with comorbidities, may be less likely to tolerate certain combination chemotherapy regimens, frequently resulting in discontinuation before the regimen is completed [16, 17]. Excess OC-related deaths are high among the elderly, who are also disproportionately affected by COVID-19. This phenomenon of excess deaths is similar to that which has been reported in patients with diabetes mellitus, chronic liver disease, and cirrhosis [7, 8, 18]. Independent risk factors for mortality include age 70 years or older, surgery for malignant disease, and major surgery [19].

The low mortality rate observed in Asian women is likely because they are typically younger at diagnosis, are more likely to present with localized disease, and are more likely to have ovarian cancer subtypes associated with favourable prognosis [20]. Black women with ovarian cancer have worse overall survival and have more comorbidity burden when beginning treatment when compared to white women with ovarian cancer [21]. However, during the pandemic, the mortality rate gap narrowed between white and black women, likely due to the rapid and significant decline in the incidence of ovarian cancer in white women. Excess deaths were higher among non-Hispanic AI/AN women (88.134%), thought to be partly related to the lack of opportunities for remote work (i.e., higher risk of COVID-19 exposure) in this population [22]. Similarly, in our previous study [8, 23] in chronic liver disease, excess deaths were high among non-Hispanic AI/AN populations due to less access to medical services.

The trend in ASMR for cervical cancer was stabilized pre-pandemic. This is attributed at least in part to lower detection of glandular cancers [24] and the relatively short time since the introduction of the HPV vaccine. During the pandemic, young women had the highest excess deaths from cervical cancer, contributing to the increase in ASMR. The recommendation for co-screening with HPV testing and cytology may have led to increased detection of early-stage cancers [25], and the increased ASMR is expected to decline as normal medical activities resume.

Different from the pattern observed with ovarian cancer and uterine cancer, women aged 45–64 years had the largest number of deaths, with the highest hysterectomy-corrected cervical cancer incidence rate [26]. The data reported herein may be an underestimate of the true excess burden in consideration of the incidence and mortality of cervical cancer corrected for number of women not at risk. Also during this time, a greater increase in the ASMR among older women was observed. There was a significant uptick in 2021 for younger and older women. This may be a result of missed screening opportunities at earlier ages and delayed diagnosis and treatment. However, the prolonged time from diagnosis to the initiation of treatment (< 6 months) showed limited negative effects on survival with early-stage female cancers [27]. Women older than 65 years, who overall are living longer, may have an even higher risk of cervical cancer associated with advanced age; vaccination is the best way to prevent cervical cancer in this and other age groups [28]. The most cost-effective strategy for prevention and detection would be to ensure adequate screening before age 65 years, and then continuing surveillance going forward for those with higher risk [28]. About 30% of excess deaths in this age group were associated with COVID-19. This phenomenon is pronounced in developed countries, as these countries have a larger population of individuals older than 65 years, and the excess number of deaths in this population is greater.

The overall GC mortality rate had generally decreased from 2010 to 2015, but experienced a distinct rise in 2016, reflecting the sustained decline in ovarian cancer and the increase in uterine corpus cancer. More importantly, uterine corpus cancer became the second leading cause of GC deaths after 2015. This trend paralleled the increase in the ageing population and in the prevalence of metabolic syndrome. The “Baby Boomers”, those who turned 49–67 years of age by 2013, are at high risk for uterine cancer as it occurs most frequently in postmenopausal women [29]. Additionally, this group has a higher average body mass index (BMI) [30], an increased use of postmenopausal oestrogen, and changing reproductive patterns (e.g., fewer births, earlier age at menarche) [31, 32]. These factors may contribute to the sharp rise in ASMR observed for uterine corpus cancer, although the study by Clarke et al. [33] indicated that this trend is driven by nonendometrioid subtypes, which are less-strongly-associated with oestrogen-related risk factors and obesity than endometrioid carcinoma. During the pandemic, CC and OC peaked in 2021, but the upward trend of UCC is likely to continue until 2040 [34].

Among elderly women, the higher observed ASMR of UCC is associated with COVID-19. In addition to the rise in all-cause mortality, obesity, and diabetes during the pandemic, it should be noted that care for the elderly with stroke or other cardiovascular events was also disrupted with an increase in incidence of these conditions [7, 35–37].

Black women are twice as likely to die as a result of uterine corpus cancer when compared to white women, and are more likely to be diagnosed with aggressive non-endometrioid subtypes [38]. The key to decreasing the incidence of uterine cancer control lies in these non-endometrioid subtypes, which are largely responsible for the rise in incidence and mortality of UCC. The racial disparity is highlighted by the gap in 5-year survival rates, with 64% of black women surviving compared to 86% of white women. Studies have shown that black Americans have a 33% higher risk of dying of cancer than white Americans, as they often receive poorer-quality treatment and have lower general access to healthcare [39]. For example, non-Hispanic white women are more likely to receive guideline-concordant treatment. Furthermore, black women experience more social, economic, and political marginalization that translates into higher allostatic load and weathering on the body [40]. Chronic stress may increase the risk of tumours driven by oestrogen, leaving black women at risk for aggressive uterine cancer as they age [31].

To our knowledge, this study represents the most comprehensive analysis of GC mortality trends and the impact of the COVID-19 pandemic to date in the U.S. However, this study has limitations. Our analyses do not account for histologic subtypes of the various types of GC presented, which have different effects on death at different age. Also, in the U.S., hysterectomy-corrected endometrial cancer incidence rates were estimated to be about 30% higher than uncorrected rates [41]. The data presented here underestimate the rate of UCC among at-risk women. Finally, we have only analysed publicly available data, which may limit interpretation; however, this potential limitation is likely minor.

## Conclusions

We found a decrease in mortality for ovarian cancer and cervical cancer, but a rise in mortality for uterine corpus cancer in the U.S. Our analysis provides strong evidence that the burden of GC is not equally distributed across age and racial groups. It is possible that long-term sequelae of delayed or deferred care due to the pandemic and of COVID-19 disease itself will result in increased mortality in future years. As such, regular preventative screening, vaccination, and treatment should be resumed as soon as possible with respect to GC. Ideally, this study will support the implementation of future policies related to GC prevention and management, with emphasis on the need for these policies to protect the most vulnerable populations.

### Electronic supplementary material

Below is the link to the electronic supplementary material.

Supplementary Material 1

## Data Availability

The NVSS can be accessed through this website: https://wonder.cdc.gov/mcd-icd10-provisional.html.

## Declarations

### Transparency statement

The lead author affirms that this manuscript is an honest, accurate, and transparent account of the study being reported; that no important aspects of the study have been omitted; and that any discrepancies from the study as planned (and, if relevant, registered) have been explained.

## Abbreviations

GC: Gynaecologic cancer
OC: Ovarian cancer
CC: Cervical cancer
UCC: Uterine corpus cancer
HPV: Human papilloma virus
ASMR: Age-standardized mortality rates
APC: Annual percentage change
COVID-19: Coronavirus Disease 2019
NVSS: National Vital Statistics System
