# Supplementary material for: Trends in gynaecologic cancer mortality and the impact of the COVID-19 pandemic in the United States
Source: Infect Agent Cancer. 2024 Feb 20;19:4. doi: 10.1186/s13027-024-00567-6 (PMC10880335; doi:10.1186/s13027-024-00567-6)

**Supplemental Online Content**

**Trends in gynaecologic cancer mortality and the impact of the COVID-19 pandemic in the United States**

Yuyan Xi,**^＃^** Yuxin Guo,**^＃^** Sikai Qiu, Fan Lv, Yujiao Deng, Jingyi Xie, Zixuan Xing, Yajing Bo, Chenyu Chang, Fan Zhang, Fanpu Ji,* Mu Li*

**Table of Content**

**Table S1.** Age-standardised mortality rate among U.S. women with gynaecologic cancer by race/ethnicity, 2010 -2020

**Table S2.** Race characteristics of gynaecologic cancer deaths and annual percent change in mortality among women with gynaecologic cancer in the U.S., 2010 -2020

**Table S3.** Age-standardised mortality rate and annual percent change in mortality in women with gynaecologic cancer in the U.S., 2010-2022

**Table S4.** Annual percentage change in mortality in women with gynaecologic cancer in the U.S. by age group, 2010 -2022

**Figure S1.** Age-standardized mortality rate for gynaecologic cancer in the U.S. in 2010-2020 by race and ethnicity. Data for racial subgroups was only available through the end of 2020, as the addition of the "more than one race" category to the CDC website in 2021. AI/AN: American Indian/Alaska Native.

**Table S1.** Age-standardised mortality rate among U.S. women with gynaecologic cancer by race/ethnicity, 2010 -2020^§^

| **Age-standardised Mortality Rate (Per 100,000 Persons)** | | | | | | |
| --- | --- | --- | --- | --- | --- | --- |
|  |  | **Pre-Pandemic Referent Epoch 2010** | **Pre-Pandemic Referent Epoch 2019** | **Pandemic Epoch 1 2020** | | |
|  | **Race/Ethnicity** |  |  | **Observed** | **Predicted [95% CI]** | **% Increase*** |
| Gynaecologic Cancer† | Hispanic | 8.638 | 8.532 | 8.653 | 8.698 [8.019-9.376] | -0.513 |
|  | Non-Hispanic AI/AN | 9.822 | 7.803 | 9.231 | 6.872 [4.334-9.410] | +34.330 |
|  | Non-Hispanic Asian | 7.400 | 7.028 | 7.822 | 6.575 [4.861-8.288] | +18.966 |
|  | Non-Hispanic Black | 13.431 | 13.646 | 13.833 | 14.071 [13.114-15.029] | -1.694 |
|  | Non-Hispanic White | 11.237 | 10.138 | 10.466 | 10.402 [9.862-10.943] | +0.620 |
| Ovarian Cancer | Hispanic | 5.152 | 4.392 | 4.128 | 4.284 [4.082 to 4.486] | -3.635 |
|  | Non-Hispanic AI/AN | 6.154 | 3.449 | 5.290 | 2.812 [0.865 to 4.759] | +88.134 |
|  | Non-Hispanic Asian | 4.477 | 3.841 | 4.105 | 3.439 [2.491 to 4.387] | +19.375 |
|  | Non-Hispanic Black | 6.666 | 5.501 | 5.469 | 5.430 [4.898 to 5.963] | +0.726 |
|  | Non-Hispanic White | 7.579 | 5.762 | 5.791 | 5.655 [5.525 to 5.786] | +2.403 |
| Cervical Cancer | Hispanic | 2.360 | 2.054 | 2.195 | 1.846 [1.533 to 2.158] | +18.883 |
|  | Non-Hispanic AI/AN | 2.494 | 2.035 | 1.928 | 2.033 [1.688 to 2.378] | -5.174 |
|  | Non-Hispanic Asian | 1.636 | 1.361 | 1.580 | 1.410 [1.185 to 1.635] | +12.059 |
|  | Non-Hispanic Black | 3.810 | 3.191 | 3.098 | 3.086 [2.703 to 3.469] | +0.378 |
|  | Non-Hispanic White | 1.839 | 1.809 | 1.880 | 1.802 [1.762 to 1.842] | +4.314 |
| Uterine Corpus Cancer | Hispanic | 1.171 | 2.116 | 2.334 | 2.412 [2.082 to 2.742] | -3.232 |
|  | Non-Hispanic AI/AN | NA | 2.384 | 2.020 | NA | NA |
|  | Non-Hispanic Asian | 1.254 | 1.826 | 2.139 | 2.059 [1.655 to 2.463] | +3.906 |
|  | Non-Hispanic Black | 2.988 | 4.936 | 5.365 | 5.529 [5.104 to 5.954] | -2.958 |
|  | Non-Hispanic White | 1.864 | 2.609 | 2.828 | 2.671 [2.314 to 3.028] | +5.887 |

NA, not applicable; AI/AN, American Indian/Alaska Native. ^§^Data for racial subgroups was only available through the end of 2020, as the addition of the "more than one race" category to the CDC website in 2021. ^†^Gynaecologic cancer includes cervix uteri (International Statistical Classification of Disease and Related Health Problems, 10th revision [ICD-10] code C53), corpus uteri (ICD-10 code C54), and ovary (ICD-10 code C56). *Denotes the % of increase from predicted to observed value.

**Table S2.** Race characteristics of gynaecologic cancer deaths and annual percent change in mortality among women with gynaecologic cancer in the U.S., 2010 -2020

|  |  | **Deaths [%]** | | | | **Average APC (95% CI) 2010–2020** | **Trend segment** | | |
| --- | --- | --- | --- | --- | --- | --- | --- | --- | --- |
|  | **Race/Ethnicity** | **2010-2021** | **2010** | **2019** | **2020** |  | **Year** | **APC (95% CI)** | ***p*-value** |
| Gynaecologic Cancer† | Hispanic | 22392 [8.05] | 1608 [6.72] | 2392 [8.98] | 2551 [9.12] | 0.0 (-0.6 to 0.6) | 2010-2020 | 0.0 (-0.6-0.6) | 0.976 |
|  | Non-Hispanic AI/AN | 1533 [0.55] | 126 [0.53] | 138 [0.52] | 168 [0.60] | -1.7 (-4.0 to 0.6) | 2010-2020 | -1.7 (-4.0-0.6) | 0.133 |
|  | Non-Hispanic Asian | 9287 [3.34] | 666 [2.78] | 992 [3.72] | 1130 [4.04] | 0.3 (-0.9 to 1.7) | 2010-2020 | 0.3 (-0.9-1.7) | 0.558 |
|  | Non-Hispanic Black | 35547 [12.78] | 2773 [11.58] | 3726 [13.98] | 3869 [13.83] | 0.1 (-0.7 to 1.0) | 2010-2015 | -1.5 (-3.0-0.0) | 0.050 |
|  |  |  |  |  |  |  | 2015-2020 | 1.8 (0.3-3.4) | 0.027 |
|  | Non-Hispanic White | 209452 [75.29] | 18764 [78.39] | 19401 [72.80] | 20251 [72.41] | -0.7 (-1.5 to 0.1) | 2010-2014 | -1.9 (-3.7-0.1) | 0.056 |
|  |  |  |  |  |  |  | 3014-2020 | 0.0 (-1.0-1.1) | 0.963 |
| Ovarian Cancer | Hispanic | 11769 [7.07] | 906 [5.83] | 1200 [8.24] | 1197 [8.08] | -2.0 (-2.6 to -1.4) | 2010-2020 | -2.0 (-2.6 to -1.4) | <0.001 |
|  | Non-Hispanic AI/AN | 866 [0.52] | 76 [0.49] | 60 [0.41] | 94 [0.63] | -3.6 (-7.1 to -0.0) | 2010-2020 | -3.6 (-7.1 to -0.0) | 0.048 |
|  | Non-Hispanic Asian | 5268 [3.16] | 400 [2.57] | 543 [3.73] | 591 [3.99] | -0.8 (-1.9 to 0.2) | 2010-2020 | -0.8 (-1.9 to 0.2) | 0.113 |
|  | Non-Hispanic Black | 15766 [9.47] | 1345 [8.65] | 1508 [10.35] | 1517 [10.23] | -2.2 (-2.8 to -1.5) | 2010-2020 | -2.2 (-2.8 to -1.5) | <0.001 |
|  | Non-Hispanic White | 132812 [79.78] | 12821 [82.46] | 11259 [77.28] | 11423 [77.07] | -2.7 (-3.0 to -2.4) | 2010-2020 | -2.7 (-3.0 to -2.4) | <0.001 |
| Cervical Cancer | Hispanic | 6542 [12.91] | 502 [11.55] | 634 [13.57] | 689 [14.05] | -0.9 (-1.6 to -0.2) | 2010-2020 | -0.9 (-1.6 to -0.2) | 0.023 |
|  | Non-Hispanic AI/AN | 399 [0.79] | 34 [0.78] | 38 [0.81] | 35 [0.71] | -2.2 (-4.1 to -0.3) | 2010-2020 | -2.2 (-4.1 to -0.3) | 0.025 |
|  | Non-Hispanic Asian | 2068 [4.08] | 153 [3.52] | 190 [4.07] | 227 [4.63] | -1.6 (-3.5 to 0.4) | 2010-2020 | -1.6 (-3.5 to 0.4) | 0.108 |
|  | Non-Hispanic Black | 9335 [18.41] | 838 [19.28] | 859 [18.38] | 856 [17.45] | -2.4 (-3.1 to -1.6) | 2010-2020 | -2.4 (-3.1 to -1.6) | <0.001 |
|  | Non-Hispanic White | 32349 [63.81] | 2819 [64.86] | 2952 [63.17] | 3098 [63.16] | -0.1 (-0.5 to 0.3) | 2010-2020 | -0.1 (-0.5 to 0.3) | 0.652 |
| Uterine Corpus Cancer | Hispanic | 4153 [6.70] | 203 [4.93] | 569 [7.58] | 669 [8.01] | 7.0** (3.7 to 10.3) | 2010-2014 | 1.0 (-6.3 to 8.9) | 0.755 |
|  |  |  |  |  |  |  | 2014-2020 | 11.1 (6.7 to 15.7) | 0.001 |
|  | Non-Hispanic AI/AN | 274 [0.44] | 16 [0.39] | 42 [0.56] | 39 [0.47] | NA | NA | NA | NA |
|  | Non-Hispanic Asian | 1977 [3.19] | 115 [2.79] | 260 [3.46] | 315 [3.77] | 4.8* (1.0 to 8.7) | 2010-2013 | -5.4 (-17.0 to 7.8) | 0.338 |
|  |  |  |  |  |  |  | 2013-2020 | 9.5 (5.7 to 13.4) | 0.001 |
|  | Non-Hispanic Black | 10556 [17.04] | 598 [14.52] | 1369 [18.24] | 1514 [18.13] | 6.1** (5.0 to 7.2) | 2010-2015 | 2.9 (1.0 to 4.8) | 0.009 |
|  |  |  |  |  |  |  | 2015-2020 | 9.4 (7.4 to 11.4) | <0.001 |
|  | Non-Hispanic White | 45003 [72.63] | 3187 [77.37] | 5266 [70.16] | 5812 [69.61] | 4.6** (2.4 to 6.7) | 2010-2013 | -1.6 (-8.5 to 5.8) | 0.609 |
|  |  |  |  |  |  |  | 2013-2020 | 7.3 (5.3 to 9.4) | <0.001 |

NA, not applicable; AP, annual percentage change; AI/AN, American Indian/Alaska Native. ^†^Gynaecologic cancer includes cervix uteri (International Statistical Classification of Disease and Related Health Problems, 10th revision [ICD-10] code C53); corpus uteri (ICD-10 code C54); and ovary (ICD-10 code C56). * Indicates that the Average APC is significantly different from zero at the alpha = 0.05 level. ** Indicates that the Average APC is significantly different from zero at the alpha = 0.001 level.

**Table S3.** Age-standardised mortality rate and annual percent change in mortality in women with gynaecologic cancer in the U.S., 2010-2022

|  | **Deaths (age-standardised rate per 100,000)** | | | | **Average APC (95% CI) 2010–2022** | **Trend segment** | | |
| --- | --- | --- | --- | --- | --- | --- | --- | --- |
|  | **2010 (Pre-pandemic referent epoch)** | **2020 (Pandemic epoch 1)** | **2021 (Pandemic epoch 2)** | **2022 (Pandemic epoch 3)** |  | **Year** | **APC (95% CI)** | **p value** |
| Gynecologic Cancer^†^ | 23937(11.11) | 27969(10.49) | 28154(10.68) | 28017(10.60) | -0.4(-0.9 to 0.1) | 2010-2014 | -2.0(-3.6 to -0.5) | 0.015 |
|  |  |  |  |  |  | 2014-2022 | 0.4(-0.1 to 1.0) | 0.1 |
| Ovarian Cancer | 15548(7.19) | 14822(5.50) | 14807(5.51) | 14597(5.48) | -2.2**(-2.7 to -1.8) | 2010-2019 | -2.8(-3.1 to -2.4) | < 0.001 |
|  |  |  |  |  |  | 2019-2022 | -0.6(-2.5 to 1.3) | 0.487 |
| Cervical Cancer | 4346(2.04) | 4905(2.03) | 5075(2.11) | 4772(1.95) | -0.2 (-1.2 to 0.8) | 2010-2018 | -0.6(-1.6 to 0.4) | 0.188 |
|  |  |  |  |  |  | 2018-2022 | 0.6(-2.2 to 3.6) | 0.627 |
| Uterine Corpus Cancer | 4119(1.90) | 8349(3.03) | 8393 (3.08) | 8787(3.21) | 4.9**(3.1 to 6.7) | 2010-2013 | -0.9 (-7.7 to 6.4) | 0.774 |
|  |  |  |  |  |  | 2013-2022 | 6.9(5.6 to 8.3) | < 0.001 |

APC, Annual percentage change. †Gynaecologic cancer includes cervix uteri (International Statistical Classification of Disease and Related Health Problems, 10th revision [ICD-10] code C53), corpus uteri (ICD-10 code C54), and ovary (ICD-10 code C56). ** Indicates that the Average APC is significantly different from zero at the alpha = 0.001 level.

**Table S4.** Annual percentage change in mortality in women with gynaecologic cancer in the U.S. by age group, 2010 -2022

|  |  |  | **Trend segment** | | |
| --- | --- | --- | --- | --- | --- |
|  |  | **Average APC(95% CI) 2010–2022** | **Year** | **APC (95% CI)** | **p value** |
| Gynecologic Cancer† | 25-44 years | 0.3(-1.4 to 1.9) | 2010-2018 | -0.4(-2.0 to 1.2) | 0.569 |
|  |  |  | 2018-2022 | 1.6(-3.0 to 6.6) | 0.449 |
|  | 45-64 years | -0.3(-0.7 to 0.0) | 2010-2022 | -0.3(-0.7 to 0.0) | 0.052 |
|  | ≥65 years | -0.5*(-1.0 to 0.1) | 2010-2014 | -2.6(-4.2 to -1.0) | 0.005 |
|  |  |  | 2014-2022 | 0.6(0.1 to 1.2) | 0.031 |
| Ovarian Cancer | 25-44 years | -1.6(-2.5 to -0.8) | 2010-2022 | -1.6(-2.5 to -0.8) | 0.002 |
|  | 45-64 years | -2.0(-2.4 to -1.7) | 2010-2022 | -2.0(-2.4 to -1.7) | < 0.001 |
|  | ≥65 years | -2.3**(-2.8 to -1.9) | 2010-2019 | -3.1(-3.4 to -2.7) | < 0.001 |
|  |  |  | 2019-2022 | -0.1(-2.1 to 1.9) | 0.888 |
| Cervical Cancer | 25-44 years | 0.0(-1.3 to 1.4) | 2010-2020 | 0.5(-0.2 to 1.2) | 0.125 |
|  |  |  | 2020-2022 | -2.3(-10.4 to 6.7) | 0.563 |
|  | 45-64 years | -0.2(-0.6 to 0.2) | 2010-2022 | -0.2(-0.6 to 0.2) | 0.242 |
|  | ≥65 years | -0.4(-1.7 to 1.0) | 2010-2019 | -1.3(-2.3 to -0.3) | 0.020 |
|  |  |  | 2019-2022 | 2.4(-3.3 to 8.3) | 0.367 |
| Uterine Corpus Cancer | 25-44 years | 7.3(3.2 to 11.6) | 2010-2022 | 7.3(3.2 to 11.6) | 0.002 |
|  | 45-64 years | 3.7*(1.6 to 5.8) | 2010-2014 | 0.5(-2.3 to 3.5) | 0.647 |
|  |  |  | 2014-2017 | 10.7(1.1 to 21.3) | 0.034 |
|  |  |  | 2017-2022 | 2.2(0.1 to 4.3) | 0.042 |
|  | ≥65 years | 4.9**(3.5 to 6.3) | 2010-2014 | -0.8(-2.7 to 1.2) | 0.336 |
|  |  |  | 2014-2017 | 11.5(4.8 to 18.6) | 0.006 |
|  |  |  | 2017-2022 | 5.7(4.2 to 7.1) | < 0.001 |

Age data is presented in years. APC, annual percentage change. ^†^Gynaecologic cancer includes cervix uteri (International Statistical Classification of Disease and Related Health Problems, 10th revision [ICD-10] code C53), corpus uteri (ICD-10 code C54), and ovary (ICD-10 code C56). * Indicates that the Average APC is significantly different from zero at the alpha = 0.05 level. ** Indicates that the Average APC is significantly different from zero at the alpha = 0.001 level.

**Figure S1.** Age-standardized mortality rate for gynaecologic cancer in the U.S. in 2010-2020 by race and ethnicity. Data for racial subgroups was only available through the end of 2020, as the addition of the "more than one race" category to the CDC website in 2021. AI/AN: American Indian/Alaska Native.


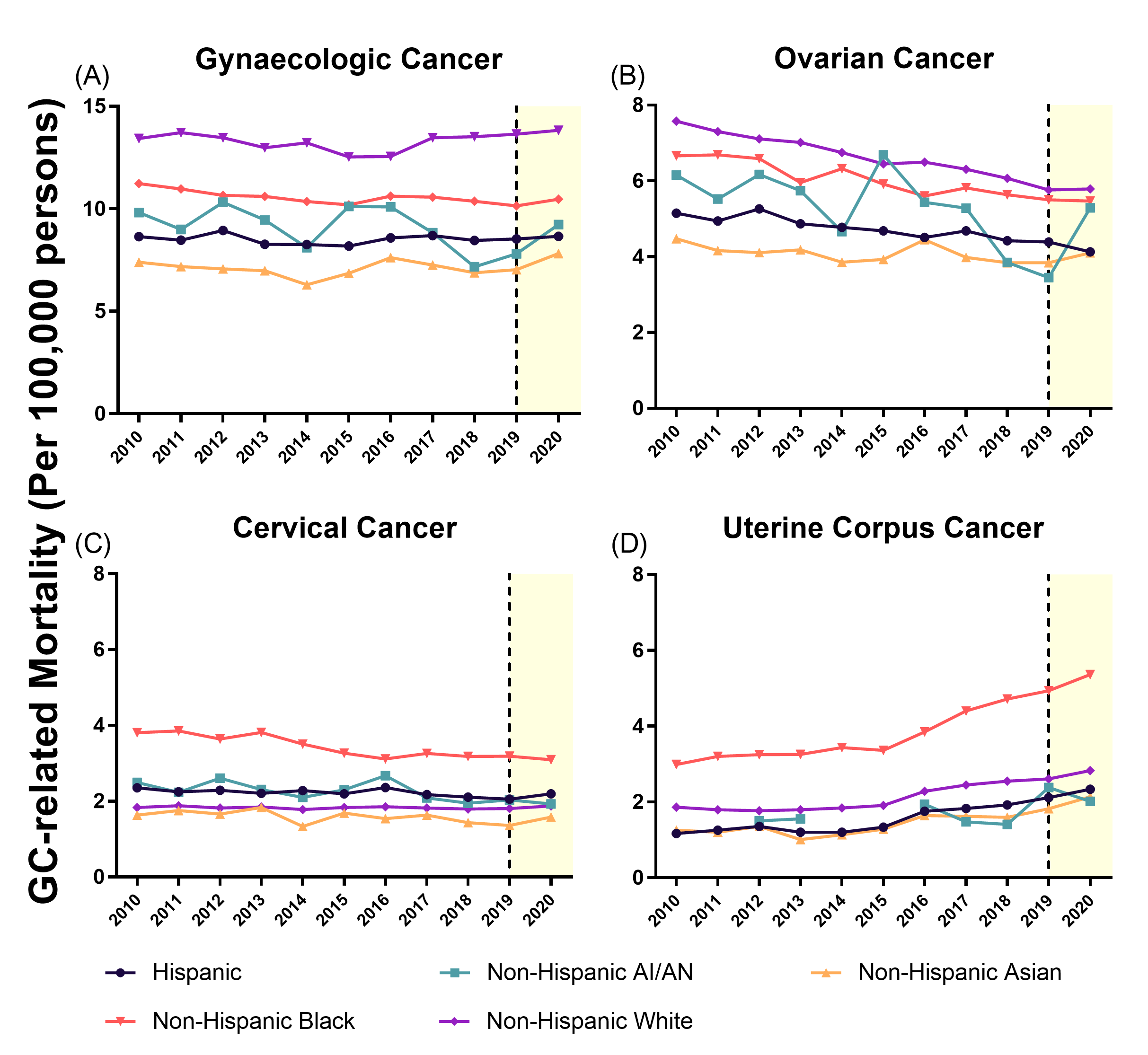

Supplement: Supplementary file 1 — Supplementary Material 1 [file 13027_2024_567_MOESM1_ESM.docx]
